# Risk-Stratified versus Cytology-Based Triage of Non-HPV 16/18-Positive for Detection of High-Grade Cervical Intraepithelial Neoplasia: Protocol of a Non- Inferiority Randomized Controlled Trial

**DOI:** 10.21203/rs.3.rs-10093990/v1

**Published:** 2026-07-25

**Authors:** Kehinde S. OKUNADE, Omolola SALAKO, Adaiah SOIBI-HARRY, Iyabo Y. ADEMUYIWA, Nosimot O. DAVIES, Fatimah M. HABEEBU-ADEYEMI, Fatimah ADEBOJE-JIMOH, Ololade ADERETI, Ayomide I. FAYINTO, Temitope V. ADEKANYE, Teniola LAWANSON, Oziegbe OGHIDE, Ayokunle M. OLUMODEJI, Hameed ADELABU, Muisi A. Adenekan, Modupe Adedeji, Adebola A. ADEJIMI, Festus O. OLOWOSELU, Matthew J. ALLSOP, Rose I. ANORLU, Jonathan S. BEREK

**Affiliations:** University of Lagos; University of Lagos; Case Western Reserve University; University of Lagos; University of Lagos; Lagos University Teaching Hospital; Lagos University Teaching Hospital; Lagos University Teaching Hospital; University of Lagos; Lagos University Teaching Hospital; University of Lagos; Lagos University Teaching Hospital; Lagos State University Teaching Hospital; University of Lagos; Lagos State University Teaching Hospital; Lagos State University Teaching Hospital; University of Lagos; University of Lagos; University of Leeds; University of Lagos; Stanford University School of Medicine

**Keywords:** Cervical cancer, HPV screening, Non-HPV16/18, Risk-stratified triage, Cytology, CIN2+, Nigeria

## Abstract

**Background:**

Human papillomavirus (HPV)-based screening is increasingly being adopted as the primary strategy for cervical cancer prevention due to its superior sensitivity compared with cytology. However, effective triage of women who test positive for high-risk HPV (hrHPV), particularly those infected with non-HPV16/18 genotypes, remains a significant challenge in low-resource settings. Cytology-based triage requires substantial laboratory infrastructure and technical expertise, which may limit its scalability. Risk-stratified triage approaches incorporating readily available clinical and demographic factors may offer a practical alternative for identifying women at highest risk of cervical precancer while reducing unnecessary referrals.

**Aim:**

The “*STRAT-CIN Trial*” aims to determine whether a risk-stratified triage algorithm is non-inferior to standard cytology-based triage for the detection of high-grade cervical intraepithelial neoplasia (CIN2+) among women positive for non-HPV16/18 high-risk HPV types.

**Methods:**

This protocol describes a two-arm, parallel, open-label, non-inferiority randomized controlled trial involving sexually active women aged 30–65 years who test positive for non-HPV16/18 high-risk HPV types during routine cervical cancer screening at the Lagos University Teaching Hospital (LUTH), Lagos, Nigeria, between June 2026 and January 2027. A total of 148 eligible women will be randomized in a 1:1 ratio to either a risk-stratified triage arm or a cytology-based triage arm. Participants in the intervention arm will be triaged using a composite algorithm incorporating age, HIV status, cigarette smoking, long-term oral contraceptive use, and multiple hrHPV genotype infections, while participants in the control arm will undergo reflex cytology according to the Bethesda 2014 classification. The primary outcome is the proportion of histologically confirmed CIN2 + lesions detected in each study arm. Secondary outcomes include diagnostic performance metrics, referral rates for colposcopy, colposcopic yield, and predictors of CIN2+. Analyses will follow the intention-to-treat principle. Differences in CIN2 + detection rates will be assessed using risk differences and corresponding 95% confidence intervals. Non-inferiority will be concluded if the lower bound of the confidence interval exceeds the pre-specified non-inferiority margin of − 10%. Logistic regression analyses will be used to identify independent predictors of CIN2+, and statistical significance will be set at P < 0.05.

**Discussion:**

The “*STRAT-CIN Trial*” will evaluate whether a simplified risk-stratified triage approach can achieve diagnostic performance comparable to conventional cytology-based triage among women with non-HPV16/18 high-risk HPV infections. By integrating easily obtainable clinical and demographic risk factors into triage decision-making, this study has the potential to reduce unnecessary colposcopy referrals, improve screening efficiency, and support the implementation of context-appropriate cervical cancer prevention strategies in resource-constrained settings. The findings will contribute important evidence toward optimizing HPV-based cervical cancer screening programs in Nigeria and other low-and middle-income countries.

**Registration:**

: PACTR202606895128487 (2nd June 2026).

## Introduction

Cervical cancer remains a major public health threat worldwide, with over 80% of cases and deaths occurring in low- and middle-income countries (LMICs) [1]. Recent global burden estimates indicate that women in low Human Development Index (HDI) regions experience nearly three times the incidence and up to six times the mortality compared to those in high-HDI countries [2, 3]. In sub-Saharan Africa, cervical cancer is among the leading causes of female cancer mortality, often affecting women during their most productive years, thereby imposing substantial socioeconomic and familial burdens [4]. In Nigeria alone, approximately 15,000 new cases are diagnosed annually, with nearly two-thirds resulting in death [5].

Persistent infection with high-risk human papillomavirus (hrHPV) is the established cause of cervical cancer [6]. As a result, HPV-based screening is increasingly being adopted as the primary screening method due to its superior sensitivity relative to cytology (Pap smear) [7, 8]. However, because HPV testing detects transient infections as well as clinically significant lesions, triage strategies for hrHPV-positive women are essential to preserve specificity and reduce unnecessary referrals. A widely adopted approach combines partial HPV genotyping—identifying HPV16/18, the types most strongly associated with cervical carcinogenesis—with reflex cytology for other high-risk types [9]. The COMPACT (COMparison of HPV genotyping And Cytology Triage) trial, for instance, demonstrated that women infected with HPV16/18 had markedly higher absolute risks of CIN2 + compared to those harbouring other hrHPV types (19.5% vs 5.6% at 12 months, respectively) [10].

Despite these advances, optimal triage strategies for women infected with non-HPV16/18 types remain poorly defined, particularly in LMIC settings. Most trials have focused primarily on HPV16/18-positive women, leaving the substantial population of women with non-16/18 hrHPV infections comparatively understudied [11]. This knowledge gap is particularly critical in sub-Saharan Africa, where non-16/18 types account for a larger proportion of hrHPV infections [12] and associated lesions than in high-income settings [11]. Furthermore, although cytology-based triage remains the conventional approach, it demands a robust cytopathology infrastructure and technical expertise that are often limited in resource-constrained environments. The potential of alternative, risk-stratified triage models—incorporating factors such as age, HIV serostatus, smoking history, long-term oral contraceptive use, or multiple hrHPV-type co-infections—to improve diagnostic efficiency without sacrificing sensitivity has not been systematically evaluated in these settings.

Given the high burden of cervical precancerous lesions, the logistical limitations of cytology, and the evolving landscape of HPV-based screening, there is a compelling need to identify triage strategies that are both effective and contextually feasible. This proposed non-inferiority randomized controlled trial, titled “*Risk-Stratified versus Cytology-Based Triage of Non-HPV16/18-Positive Women* (*STRAT-CIN Trial*)”, is designed to address this evidence gap. The primary objective is to determine whether a risk-stratified triage algorithm is non-inferior to standard cytology-based triage in detecting high-grade cervical intraepithelial neoplasia (CIN2+) among women positive for non-HPV16/18 high-risk types. *The central hypothesis is that, among non-HPV16/18-positive women, the risk-stratified triage approach will achieve diagnostic sensitivity comparable to cytology while reducing unnecessary colposcopy referrals*. The secondary objectives are to (1) compare referral rates, colposcopic yield, and diagnostic performance metrics between the two triage strategies, and (2) identify key clinical and demographic predictors of CIN2 + among women infected with non-HPV16/18 hrHPV types.

## Methodology

### Study Design and Settings:

This study (*STRAT-CIN Trial*) proposes a two-arm, parallel, open-label, non-inferiority randomized controlled trial among sexually-active women at the Cytology and Colposcopy clinics of the Lagos University Teaching Hospital (LUTH), Nigeria, between June 2026 and January 2027. LUTH is the foremost healthcare institution in Lagos and serves as a referral centre for other government-owned and private hospitals in Lagos and its neighbouring states. The hospital provides a variety of care, including integrated gynecologic oncology prevention services such as Pap smearing, HPV testing and colposcopy services, including biopsy and histology. Laboratory analyses will be performed at the Department of Anatomic and Molecular Pathology (AMPATH), College of Medicine, University of Lagos, equipped with facilities for cytology, histopathology, and HPV genotyping [13].

### Participants:

The study population will consist of women aged 30–65 years who are hrHPV-positive for non-HPV16/18 types following primary HPV DNA testing during routine cervical cancer screening. Inclusion criteria will include laboratory confirmation of hrHPV infection with one or more non-HPV16/18 types (e.g., 31, 33, 35, 39, 45, 51, 52, 56, 58, 59, 66, or 68) using the validated Xpert^®^ HPV platform, age 30–65 years, and willingness to provide written informed consent and comply with study follow-up. Exclusion criteria will include the detection of HPV16 and/or HPV18 infection (triaged per standard-of-care protocols), previous diagnosis of CIN2+, cervical cancer, or history of hysterectomy, current pregnancy, immunosuppression unrelated to HIV, or prior HPV vaccination, and inability to provide informed consent or complete study procedures.

### Study endpoints:

The primary endpoint is the proportion of histologically confirmed CIN2 + lesions in each study arm, and the secondary endpoints are (1) diagnostic performance metrics (sensitivity, specificity, PPV, NPV) of the risk-stratified algorithm versus cytology for CIN2 + detection; (2) referral rates for colposcopy following triage in each arm; (3) colposcopic yield, defined as the proportion of referred women with histologically confirmed CIN2+; and (4) clinical and demographic predictors of CIN2+.

### Sample size calculation:

Sample size was calculated based on the primary objective of testing the non-inferiority of the risk-stratified algorithm compared with cytology-based triage for detecting CIN2+. Assuming a CIN2 + detection rate of 94% for cytology-based triage [14], a non-inferiority margin of 10%, a one-sided alpha of 5%, a power of 80%, and accounting for a 5% non-response or missing data rate, we estimate that n = 148 non-16/18 HPV-positive women (74 per arm) will be required to detect non-inferiority. This sample size will also provide > 80% power to detect a 20% relative reduction in unnecessary referrals (secondary endpoint) between study arms.

### Study procedures, randomization, and blinding:

**Figure 1:**
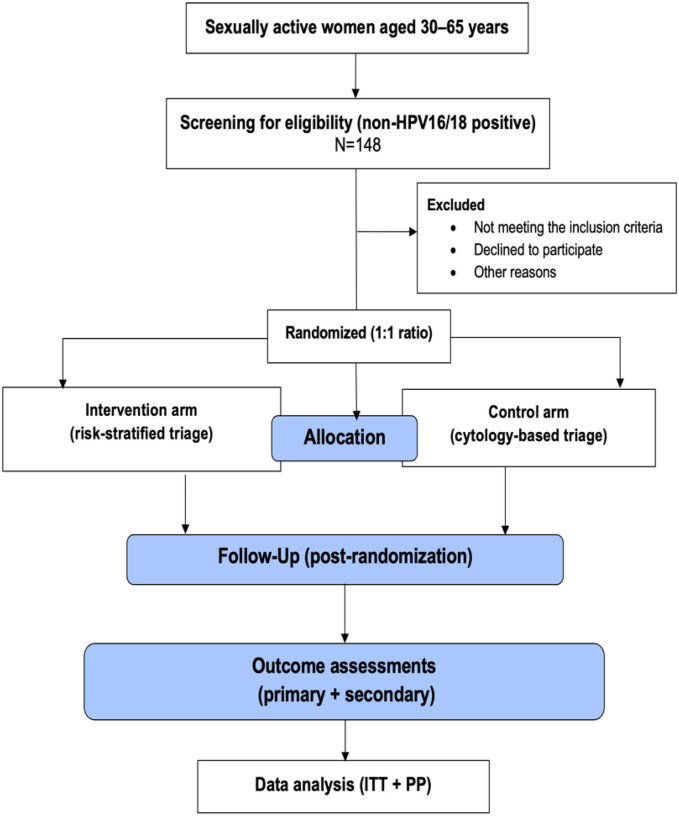
Trial participants’ flow chart

The study investigators will screen and identify eligible non-HPV16/18-positive women for the study. Eligible women will be invited to participate in the study after receiving a comprehensive explanation of its purpose, procedures, potential risks and benefits, and will provide written informed consent before enrolment. Once consent is obtained, an interviewer-administered questionnaire will be applied to obtain baseline information on sociodemographic characteristics, reproductive and sexual history, and HIV and HPV status using the electronic case report form (eCRF) on the REDCap database hosted on a secure CMUL server with audit trails and user authentication. Eligible participants will then be randomized 1:1 using a computer-generated permuted block randomization sequence (block sizes of 4–6) stratified by age (< 35 vs ≥ 35 years) and HIV status [Figure 1]. Allocation concealment will be maintained using sequentially numbered, opaque, sealed envelopes prepared and safeguarded by an independent trial statistician.

- **Intervention arm (risk-stratified triage)**: Participants will undergo triage using a risk-stratified algorithm incorporating a composite of readily available clinical and demographic data, including older age ≥ 35 years, HIV seropositivity with CD4 < 200 cells/mm^3^, cigarette smoking, long-term oral contraceptive use > 5 years, and multiple hrHPV genotype infections, to identify women at the highest risk of CIN2+ [15, 16]. Women with any of these risk factors will be referred for colposcopy, while those without will undergo repeat testing at 12 months.
- **Control arm (cytology-based triage)**: Participants will undergo reflex cytology (Pap test) interpreted per the Bethesda 2014 classification [17]. Women with atypical squamous cells of undetermined significance (ASC-US) or higher will be referred for colposcopy; those with normal cytology will undergo repeat testing at 12 months.

Colposcopy and biopsy will be performed for all screen-positive women in both arms. Histopathologic diagnosis (CIN1, CIN2, CIN3, or carcinoma) will be confirmed by two independent pathologists blinded to group allocation. Blinding of participants and clinicians is not feasible due to the procedural nature of triage methods; however, laboratory personnel and outcome assessors (cytologists, pathologists, and data analysts) will remain blinded to group allocation.

### Statistical analysis plan:

All analyses will follow the intention-to-treat (ITT) approach, supplemented by per-protocol sensitivity analyses. Descriptive statistics will summarise participant characteristics. The primary endpoint (CIN2 + detection) will be compared between arms using the proportional difference with corresponding 95% confidence intervals (CI). Non-inferiority will be concluded if the lower bound of the 95% CI for the difference (risk-stratified – cytology) is above the pre-specified non-inferiority margin (–10%). Referral rates, colposcopic yield and diagnostic performance metrics will be compared using chi-square or Fisher’s exact tests. ROC curve analysis will assess the discriminative performance of the triage methods. Logistic regression will be used to identify independent predictors of CIN2+, adjusting for age, HIV status, parity, and other covariates. Missing data will be handled using multiple imputation, assuming data are missing at random [18]. All analyses will be conducted using Stata version 18 (StataCorp LLC, Texas, USA).

### Ethical considerations:

This study (*STRAT-CIN)* will comply with the ethical principles outlined in the Declaration of Helsinki, ICH-GCP (E6[R2]), and the National Code of Health Research Ethics of Nigeria. Ethical approval was obtained from the Health Research Ethics Committee of Lagos University Teaching Hospital (LUTH-HREC – ADM/DCST/HREC/APP/8177; May 20, 2026).

Written informed consent will be obtained from all participants by the study investigators or trained clinical research assistants before any study-related procedures are undertaken. All data generated during the study will be maintained in strict confidence, securely managed, and accessed only by authorized study personnel exclusively for research purposes. The trial and statistical analysis will be conducted with fidelity, and the reporting will adhere to the Standard Protocol Items: Recommendations for Interventional Trials (SPIRIT) guidelines.

## Quality control and data monitoring

To ensure the integrity, accuracy, reliability, and validity of study data, all investigators and research personnel will undergo comprehensive pre-study training, including Good Clinical Practice (GCP) certification. The training will cover study objectives, eligibility criteria, participant recruitment, informed consent processes, data collection methods, questionnaire administration, and protocol compliance requirements. Identifiable participant information will be collected and securely entered into an electronic database by trained research staff at the study site. The database will be housed within a secure, password-protected environment, and access to identifiable data will be restricted to the Principal Investigator (KSO) and other authorized personnel as required for study conduct. All study data will be handled in accordance with applicable data protection and confidentiality guidelines. Independent monitoring of the study will be conducted by designated data officers from the Centre for Clinical Trial Research and Implementation Science (CCTRIS), College of Medicine, University of Lagos. These monitors will operate independently of the study team and will perform regular monitoring visits throughout the study period to ensure adherence to the study protocol, regulatory requirements, and Good Clinical Practice standards. Monitoring activities will include verification of source documents, review of informed consent records, participant enrolment logs, case report forms, protocol deviations, and data quality checks. The monitors will ensure that study documentation is complete, accurate, and consistent with study procedures, thereby maintaining the overall quality and credibility of the research findings.

## Community engagement plan

For the proposed trial, we will implement a comprehensive community engagement strategy spanning the pre-trial, trial implementation, and post-trial phases to promote meaningful stakeholder participation, enhance trust, facilitate participant recruitment and retention, and support the translation of research findings into practice.

- Pre-trial engagement: We will identify and engage key community stakeholders, including community-based organizations (CBOs), women's health advocacy groups, patient support networks, and local community leaders. A Community Advisory Board (CAB) will be established, comprising representatives from relevant CBOs, women's groups, the Society of Gynaecology and Obstetrics of Nigeria (SOGON), the Nigerian Medical Association (NMA), community leaders, and cervical cancer survivors or former patients. The CAB will provide guidance on study design, informed consent materials, participant recruitment strategies, and culturally appropriate implementation approaches. In collaboration with participating health facilities and community partners, we will organize community sensitization and educational forums to introduce the study objectives, procedures, potential benefits and risks, while addressing misconceptions and concerns about participation in clinical research.
- During trial implementation: Throughout the study, we will maintain continuous engagement with participants and community stakeholders through regular meetings with the CAB to review study progress, obtain feedback, identify emerging concerns, and strengthen community trust. Trained community health workers will serve as participant liaisons, providing education, appointment reminders, navigation support, and assistance in overcoming barriers to participation, including transportation challenges and family-related concerns. Participant information materials will be translated into appropriate local languages and supplemented with culturally sensitive visual aids to enhance comprehension and ensure inclusiveness across diverse literacy levels.
- Post-trial engagement: Following study completion, we will disseminate the study findings to participants, community members, healthcare providers, policymakers, and other stakeholders using accessible and culturally appropriate formats, including community dissemination meetings, town hall forums, policy dialogues, and easy-to-understand infographic summaries. We will also work closely with community representatives, professional associations, and local health authorities to identify opportunities for integrating successful interventions, lessons learned, and evidence generated by the trial into routine clinical practice, health programmes, and future policy and advocacy initiatives, thereby promoting sustainability and long-term public health impact.

## Dissemination of trial information

The trial will be conducted and reported in accordance with the Consolidated Standards of Reporting Trials (CONSORT) guidelines to ensure transparency, completeness, and methodological rigour. Study findings will be disseminated through publication in peer-reviewed scientific journals and presentations at relevant scientific conferences. Any substantial amendments to the study protocol will be promptly communicated to the funding agency, study investigators, the LUTH-HREC, trial participants (where applicable), and the clinical trial registry in accordance with regulatory and ethical requirements.

## Study Status

At the time of manuscript submission, participant recruitment for the trial has not yet commenced. Enrollment is planned to begin in July 2026, with recruitment expected to be completed by January 2027, when the final participant will be enrolled. This manuscript describes Version 4.0 of the study protocol, dated 17 June 2026.

## Discussion

Persistent infection with hrHPV is the established cause of cervical cancer [6]. As a result, HPV-based screening is increasingly being adopted as the primary screening method due to its superior sensitivity relative to cytology [7, 8]. However, because HPV testing detects transient infections as well as clinically significant lesions, triage strategies for hrHPV-positive women are essential to preserve specificity and reduce unnecessary referrals. A widely adopted approach combines partial HPV genotyping—identifying HPV16/18, the types most strongly associated with cervical carcinogenesis—with reflex cytology for other high-risk types [9]. This protocol, therefore, describes a non-inferiority randomized controlled trial, titled “*Risk-Stratified versus Cytology-Based Triage of Non-HPV16/18-Positive Women* (*STRAT-CIN Trial*)”, designed to determine whether a risk-stratified triage algorithm is non-inferior to standard cytology-based triage in detecting CIN2 + among women positive for non-HPV16/18 high-risk types, and also compare referral rates, colposcopic yield, and diagnostic performance metrics between the two triage strategies in women infected with non-HPV16/18 hrHPV types. This study, therefore, introduces an innovative risk-stratified triage algorithm for women positive for non-HPV16/18 high-risk types, a population often overlooked in existing screening models. Unlike conventional cytology-based triage, which depends on resource-intensive laboratory infrastructure, the proposed algorithm will integrate easily obtainable clinical and demographic risk factors into REDCap to guide colposcopy referral decisions. By adopting a non-inferiority trial design, the study will rigorously test whether this simplified, data-driven approach can maintain diagnostic accuracy while reducing unnecessary referrals. This innovation aligns with the WHO’s call for scalable, context-appropriate screening strategies in low-resource settings [19] and has the potential to transform cervical cancer prevention in sub-Saharan Africa.

## Supplementary Material

Supplementary Files

This is a list of supplementary files associated with this preprint. Click to download.

- SPIRITChecklist.docx
- CONSENTFORM.docx
- SupplementaryFileCRF.docx

## Data Availability

No datasets will be generated or analysed during the current study, as this article only describes the study protocol. Upon completion of the study, the authors intend to make the full study protocol, deidentified participant-level dataset, and statistical analysis code publicly available
